# Development and validation of a nomogram to predict B-cell primary thyroid malignant lymphoma-specific survival: A population-based analysis

**DOI:** 10.3389/fendo.2022.965448

**Published:** 2022-10-11

**Authors:** Shuai Jin, Lang Xie, Yanwei You, Chengli He, Xianghai Li

**Affiliations:** ^1^ School of Big Health, Guizhou Medical University, Guiyang, China; ^2^ Hospital Infection Management Department, First People's Hospital of Bijie City, Bijie, China; ^3^ Division of Sports Science & Physical Education, Tsinghua University, Beijing, China; ^4^ School of Clinical Medicine, Guizhou Medical University, Guiyang, China; ^5^ Department of Clinical Chinese Medicine, The Affiliated Hospital of Guizhou Medical University, Guiyang, China

**Keywords:** B cell primary thyroid malignant lymphoma, predictive model, SEER database, Cox model, cancer-specific survival

## Abstract

B cell primary thyroid malignant lymphoma (BC-PTML) accounts for 95% of all cases of PTML. However, development of effective treatment and management strategies for BC-PTML is challenging owing to the rarity of this disease. This study assessed data from 1,152 patients in the Surveillance, Epidemiology, and End Results (SEER) database who were diagnosed with BC-PTML during 2000–2015. Patients were randomly divided into a training group (n=806) and a test group (n=346) at a ratio of 7:3 using the hold-out method. Kaplan-Meier analysis and log-rank tests were used to calculate the survival rate of patients. Subsequently, a stepwise Cox regression model was established to screen the prognostic factors of patients with BC-PTML, and these variables were used to construct a nomogram to predict 5-, 10-, and 15-year BC-PTML cancer-specific survival (CSS). The discrimination and calibration of the new model were evaluated using the concordance index (C-index) and calibration curves, and the accuracy and benefits of the model were assessed through comparison with the traditional Ann Arbor staging system using decision curve analysis (DCA). After stepwise regression, the optimal model included radiotherapy, primary site surgery, Ann Arbor Stage, chemotherapy, histological subtype, and age at diagnosis. The C-index, area under the receiver operating characteristic curve, and DCA suggested that the nomogram had improved discriminatory ability and clinical benefit compared with the Ann Arbor staging system. In summary, this study established an effective nomogram to predict CSS in patients with BC-PTML and assist clinicians in developing effective individualized treatment strategies.

## Introduction

Primary thyroid malignant lymphoma (PTML) is a rare malignant tumor originating in the lymphoid tissue of the thyroid gland, with an incidence of 2 cases per 1 million individuals. PTML accounts for 0.6–5% of all thyroid cancers and approximately 2.5% of systemic malignant lymphomas (1). The typical clinical presentation of this disease is a tough, lobulated mass in the thyroid gland that grows rapidly over a short period and may be accompanied by hoarseness, breathlessness, and symptoms such as fever, night sweats, and weight loss. A previous history of Hashimoto’s thyroiditis is a recognized risk factor for PTML (2).

B cell primary thyroid malignant lymphoma (BC-PTML) accounts for >95% of all cases of PTML (3). Of these, diffuse large B-cell lymphoma (DLBCL) accounts for 50–80% of cases while mucosa-associated lymphoid tissue (MALT) lymphoma accounts for 20–30%. Patients with MALT tend to have a better prognosis compared with those with DLBCL (4). Development of effective treatments and management strategies for BC-PTML is challenging owing to the rarity of this cancer.

Nomograms are convenient and accessible tools for disease prognosis that have been used to predict cancer outcomes (5). They provide a visual analysis of patient prognosis using known and significant predictive variables and calculate individualized survival probabilities. Ann Arbor staging is a tool commonly used to predict the progression of malignant lymphoma and design treatment strategies (6). However, the Ann Arbor staging method only considers the properties of the tumor and does not take into account patient characteristics. The current study aimed to establish a nomogram for BC-PTML to better characterize the prognostic factors associated with this disease. A PTML dataset from the Surveillance, Epidemiology, and End Results (SEER) database was used to create a cancer-specific survival (CSS) prognostic nomogram for patients with BC-PTML. The ability of the nomogram to predict BC-PTML patient outcomes was compared with the Ann Arbor staging system.

## Materials and methods

### Patient selection and data processing

**Figure 1:**
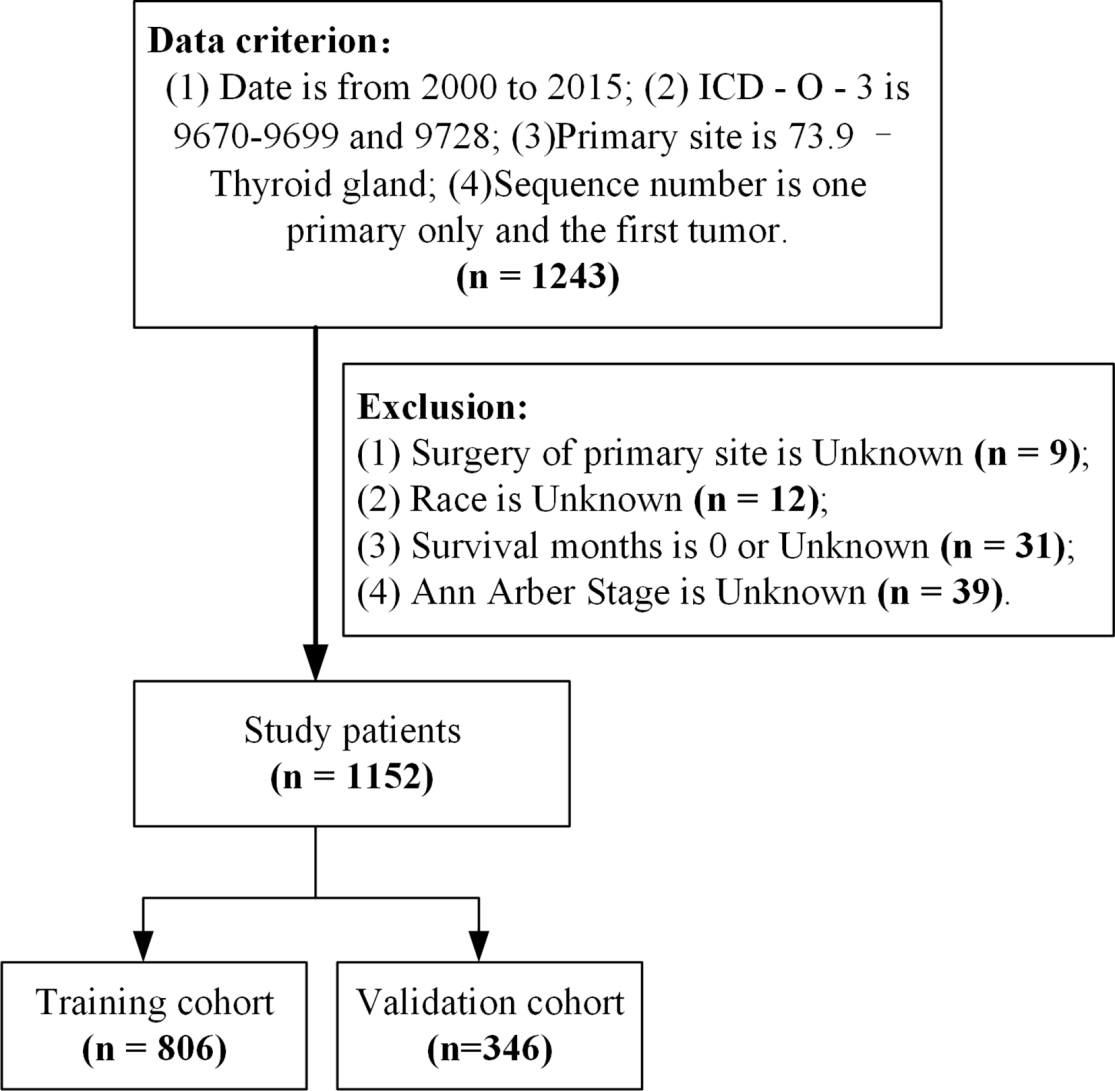
Sample selection process flowchart. ICD-O-3, International Classification of Diseases for Oncology (Third Edition).

Study subject data were downloaded from the official website of the SEER database (https://seer.cancer.gov/, covering 18 registries including samples from 2000–2018) using SEER*Stat (Version 8.3.9.2, https://seer.cancer.gov/data-software/). Samples were included if they (1) were from 2000–2015 (because Ann Arbor staging was not updated after 2016) (2), had an ICD-O-3 (International Classification of Diseases for Oncology, Third Edition) code 9670 to 9699 or 9728 (7, 8), (3) had a primary site code of 73.9 for thyroid gland, and (4) the sequence number indicates the only primary or first primary tumors. Samples were excluded if (1) the primary site surgery was unknown, (2) race of the patient was unknown, (3) survival month was 0 or unknown, or (4) Ann Arbor Stage was unknown (**Figure 1**). A total of 1,152 samples from patients with BC-PTML were included in the final cohort.

This study included BC-PTML patient demographics (age at diagnosis, race, sex, marital status), tumor characteristics (histological subtype, Ann Arbor Stage), treatment (primary site surgery, radiation, chemotherapy), follow-up time (survival months), and cancer-specific survival (CSS)—a total of 11 variables. BC-PTML was classified as diffuse large B cell lymphoma-NOS (DLBCL), lymphoma of the extra-nodal marginal zone of mucosa-associated lymphoid tissue (MALT), and other subtypes of BC-PTML. The study outcome was CSS in BC-PTML patients. Since the SEER database is publicly available to researchers and no personal patient information was included in the analysis, ethical clearance was not required for this study.

### Nomogram development and analyses

To construct and validate the nomogram model, the 1,152 patients were randomized into a training cohort (n=806) and a validation cohort (n=346) at a ratio of 7:3 (9, 10). The training cohort was used to construct the nomogram and the validation cohort was used to test the performance of the model built from the training set. Stepwise regression based on the Akaike information criterion (AIC) minimum was used to select variables for the nomogram (11). Kaplan-Meier (KM) and log-rank tests were employed to compare differences in cumulative survival within each group and univariate and multivariate Cox proportional risk models were used to screen for independent prognostic factors. The optimal model for multi-factor analysis was selected using stepwise regression, and the model was optimal when the AIC was minimal. Optimal cutoff values for the survival analysis of each measure were calculated with X-tile software (https://medicine.yale.edu/lab/rimm/research/software/). Statistical differences in age distribution between the training and validation cohorts were evaluated using the Chi-square test.

The nomogram was used to assess the CSS of BC-PTML patients at 5-, 10-, and 15-years. The consistency index (C-index), calculated using the bootstrapping (n=1000) method, and the area under the working characteristic curve (AUC) of the subjects over time were used to assess the identification ability of the model (12). Calibration plots were used to evaluate calibrating ability of the model (13). C-index and AUC values range from 0.5 to 1, with 0.5 indicating that a model lacks discrimination and 1 indicating that there is perfect discrimination; values >0.7 suggest that a model has strong discrimination. Decision curve analysis (DCA) was used to evaluate the clinical benefit and application value of the nomogram (14). R (https://www.r-project.org/) was used for all statistical analyses and plot creation. *P*-values were tested using a two-sided test, and *P <*0.05 was considered statistically significant.

## Results

### Patient and disease characteristics

A total of 1,152 patients who were diagnosed with BC-PTML from 2000 to 2015 were included in the study. Using a leave-out method, patients were randomly split into training and validation cohorts at a 7:3 ratio, with 806 and 346 patients in each group, respectively. The mean survival time for patients with BC-PTML was 191 months, more than 90% of patients were <80 years of age, there were almost twice as many females as males, and DLBCL and MALT subtypes accounted for 63.7% and 20.3% of the samples, respectively. At the time of diagnosis, 56% of patients were married, and approximately 16% were widowed. The tumors in the majority of patients were classified as Ann Arbor Stage I and II. Most patients underwent surgical treatment and chemotherapy (>60%), and 47% received radiotherapy. Patients in the training and validation cohorts were comparable as confirmed by Chi-square analysis (**Table 1**).

**Table 1:** Characteristics of BC-PTML patients included in the study and analysis of differences.

Characteristic	Total patients[n (%)]	Training cohort[n (%)]	Validation cohort[n (%)]	*P*-value
**Age (years)**				0.712
13–64	588 (51.0)	409 (50.7)	179 (51.7)	
65–80	427 (37.1)	297 (36.8)	130 (37.6)	
81–98	137 (11.9)	100 (12.4)	37 (10.7)	
**Race**				0.135
Black	25 (2.2)	13 (1.6)	12 (3.5)	
Other	102 (8.9)	73 (9.1)	29 (8.4)	
White	1025 (89.0)	720 (89.3)	305 (88.2)	
**Sex**				0.020
Female	783 (68.0)	531 (65.9)	252 (72.8)	
Male	369 (32.0)	275 (34.1)	94 (27.2)	
**Histology**				0.805
DLBCL	734 (63.7)	516 (64.0)	218 (63.0)	
MALT	234 (20.3)	165 (20.5)	69 (19.9)	
Other	184 (16.0)	125 (15.5)	59 (17.1)	
**Marital Status**				0.382
Married	648 (56.3)	464 (57.6)	184 (53.2)	
Other	321 (27.9)	217 (26.9)	104 (30.1)	
Widowed	183 (15.9)	125 (15.5)	58 (16.8)	
**Ann Arbor Stage**				0.568
Stage I	596 (51.7)	416 (51.6)	180 (52.0)	
Stage II	409 (35.5)	282 (35.0)	127 (36.7)	
Stage III	47 (4.1)	32 (4.0)	15 (4.3)	
Stage IV	100 (8.7)	76 (9.4)	24 (6.9)	
**Surgery**				0.119
No	459 (39.8)	333 (41.3)	126 (36.4)	
Yes	693 (60.2)	473 (58.7)	220 (63.6)	
**Radiation**				0.700
No/Unknown	616 (53.5)	428 (53.1)	188 (54.3)	
Yes	536 (46.5)	378 (46.9)	158(45.7)	
**Chemotherapy**				0.646
No/Unknown	364 (31.6)	258 (32.0)	106 (30.6)	
Yes	788 (68.4)	548 (68.0)	240 (69.4)	
**Mean follow-up time** [Months, (95% confidence interval)]	190.94 (185.33–196.54)	190.81 (182.43–199.18)	191.40 (186.72–196.07)	0.766

### Nomogram variable screening

**Figure 2:**
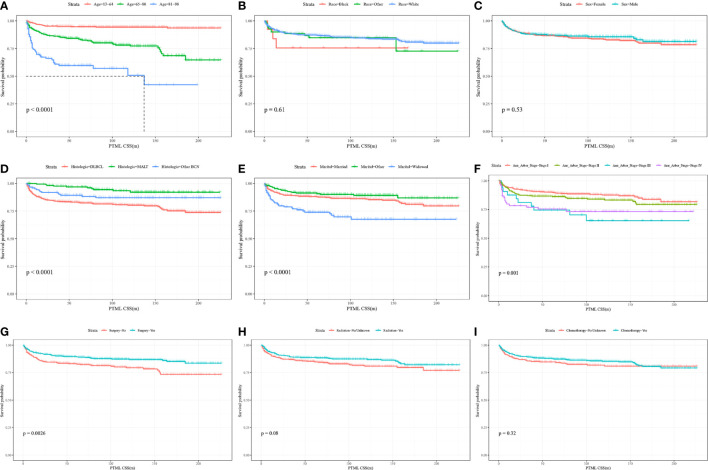
Kaplan-Meier curves of BC-PTML-specific survival in patients with age at diagnosis **(A)**, race **(B)**, sex **(C)**, histologic type **(D)**, marital status **(E)**, Ann Arbor Stage **(F)**, surgery of thyroid **(G)**, radiation **(H)** and chemotherapy **(I)**.

KM curves and log-rank tests were used to analyze the variability of CSS rates for each variable. While survival rates differed by age, histological subtype, marital status at diagnosis, Ann Arbor Stage, and primary site surgery (**Figures 2A, D–G**), cumulative survival rates did not differ by race, gender, radiotherapy, and chemotherapy (**Figures 2B, C, H, I**). (**Figure 2**). The univariate Cox model showed that age at diagnosis, histological subtype, marital status, Ann Arbor Stage, and primary site surgery were significant prognostic indicators and the multivariate model showed that age at diagnosis, histological subtype, Ann Arbor Stage, and chemotherapy were independent prognostic factors. After stepwise regression, the model with the smallest AIC when radiotherapy, primary site surgery, Ann Arbor Stage, chemotherapy, histological subtype, and age at diagnosis were included, was considered the optimal model (**Table 2**).

**Table 2:** Univariate and multivariate Cox regression analysis of BC-PTML-specific survival (Training Cohort).

Characteristic	Univariate analysis	*P*-value	Multivariate analysis	*P*-value
	HR (95% CI)		HR (95% CI)	
**Age (years)**				
13–64	Reference		Reference	
65–80	4.56 (2.82–7.35)	<0.001	4.05 (2.47–6.61)	<0.001
81–98	12.08 (7.15–20.4)	<0.001	9.89 (5.58–17.53)	<0.001
**Race**				
Black	Reference		Reference	
Other	0.61 (0.17–2.2)	0.454	0.76 (0.21–2.79	0.676
White	0.57 (0.18–1.79)	0.335	0.59 (0.18–1.90)	0.376
**Sex**				
Female	Reference		Reference	
Male	0.89 (0.61–1.29)	0.535	1.08 (0.72–1.61)	0.703
**Histology**				
DLBCL	Reference		Reference	
MALT	0.28 (0.14–0.53)	<0.001	0.23 (0.11–0.48)	<0.001
Other	0.56 (0.33–0.97)	0.039	0.54 (0.31–0.96)	0.037
**Marital Status**				
Married	Reference		Reference	
Other	0.70 (0.43–1.13)	0.143	0.76 (0.47–1.24)	0.272
Widowed	2.29 (1.52–3.46)	<0.001	1.08 (0.69–1.70)	0.742
**Ann Arbor Stage**				
Stage I	Reference		Reference	
Stage II	1.31 (0.88–1.96)	0.182	1.41 (0.93–2.13)	0.110
Stage III	2.71 (1.38–5.33)	0.004	2.54 (1.26–5.12)	0.009
Stage IV	2.36 (1.39–3.99)	0.001	1.74 (1.01–2.98)	0.045
**Surgery**				
No	Reference		Reference	
Yes	0.59 (0.41–0.83)	0.003	0.74 (0.51–1.08)	0.120
**Radiation**				
No/Unknown	Reference		Reference	
Yes	0.73 (0.51–1.04)	0.081	0.73 (0.50–1.07)	0.106
**Chemotherapy**				
No/Unknown	Reference		Reference	
Yes	0.83 (0.57–1.2)	0.317	0.47 (0.30–0.73)	<0.001

### Nomogram construction and validation

**Figure 3:**
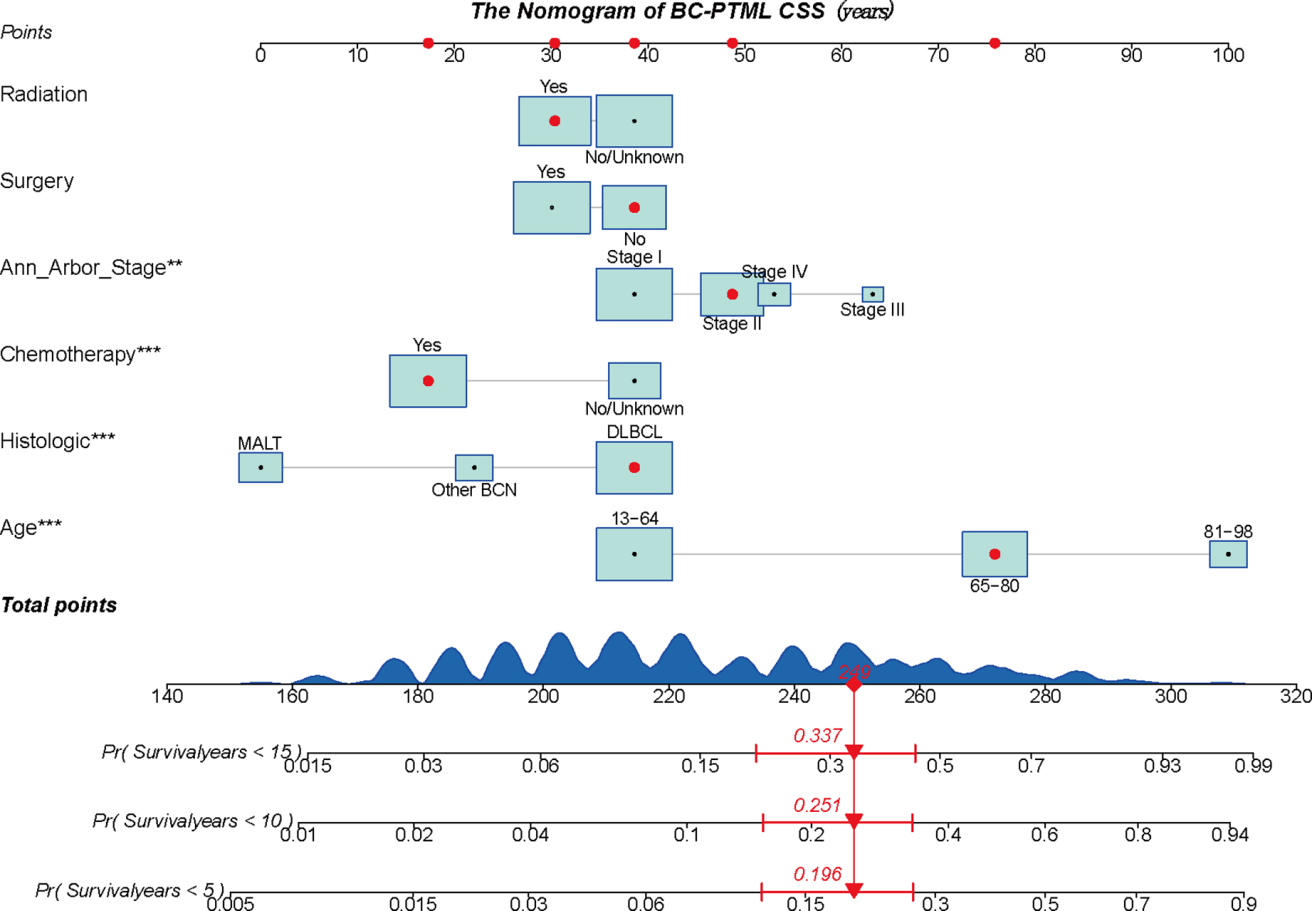
A constructed nomogram to predict the outcome of one patient with BC-PTML. The patient was 66 years of age, white, female, diagnosed with DLBCL, married, classified as Ann Arbor Stage II, did not have surgery, and did receive chemotherapy and radiotherapy. The importance of each variable was ranked according to the standard deviation along the nomogram scales. To use the nomogram, the specific points (black dots) of individual patients are located on each variable axis. The sum (n=249) of these points is located on the Total Points axis, and a line is drawn downward to the survival axis to determine the probability of 5-year (80.4%), 10-year (74.9%), and 15-year (66.3%) overall survival. BC-PTML, B cell primary thyroid malignant lymphoma. ** < 0.01, *** < 0.001.

**Figure 4:**
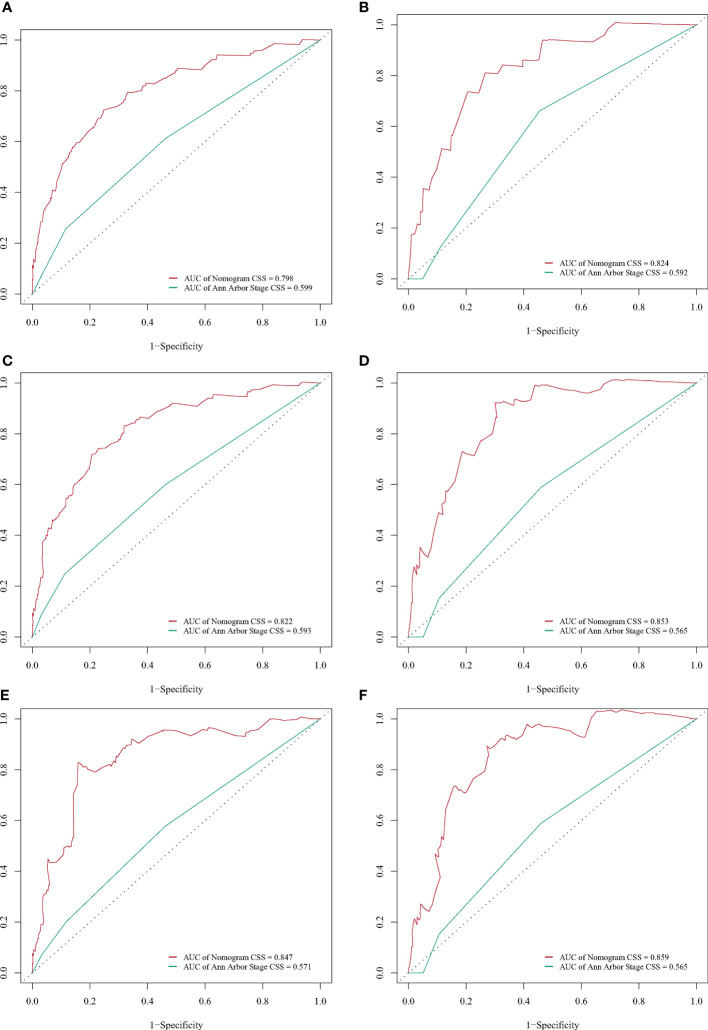
Receiver operating characteristic (ROC) curves for predicting BC-PTML-specific survival at 5-, 10-, and 15-years in the training **(A, C, E)** and validation **(B, D, F)** cohorts.

**Figure 5:**
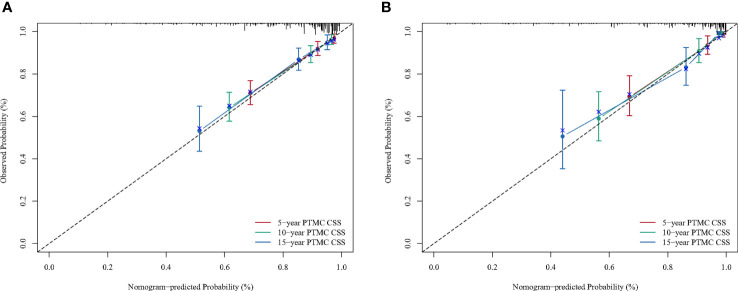
Calibration plots for predicting BC-PTML-specific survival at 5-, 10-, and 15-years in the training **(A)** and validation **(B)** cohorts.

A BC-PTML nomogram was constructed using the variables screened by the optimal model. The variables were ranked according to the standard deviation (SD) of the scales in the nomogram model (**Figure 3**). The nomogram-based C-index (training cohort = 0.777, validation cohort = 0.762) was higher compared with that of the Ann Arbor staging system (training cohort = 0.622, validation cohort = 0.585). In addition, compared with the Ann Arbor staging system, the nomogram model had improved discrimination in the training (**Figures 4A, C, E**) and validation (**Figures 4B, D, F**) groups at predicting the 5-, 10-, and 15-year CSS of patients with BC-PTML. Calibration plots of the nomogram showed good agreement between actual observations and predictions of 5-, 10-, and 15-year CSS of patients with BC-PTML in the training and validation groups (**Figure 5**).

**Figure 6:**
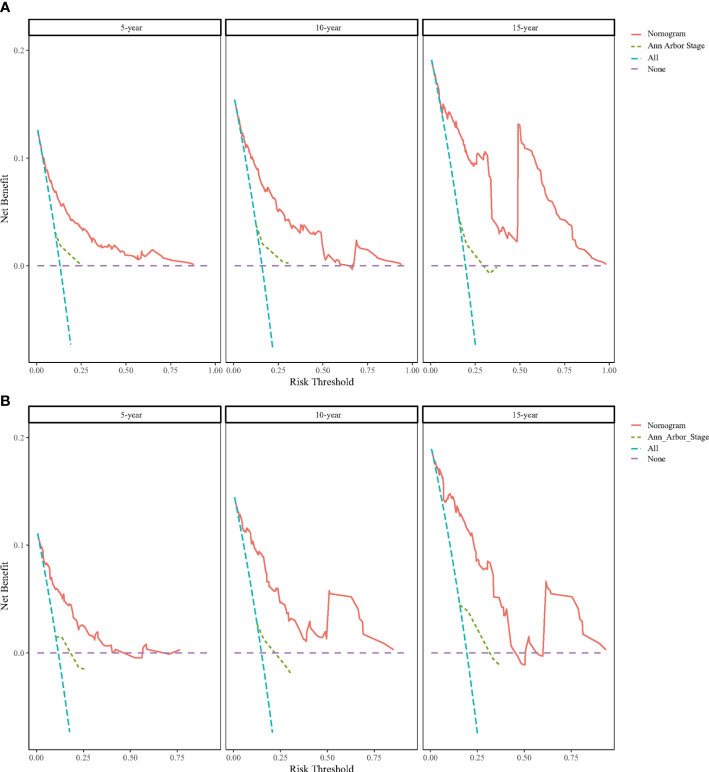
Decision curve analysis (DCA) for predicting BC-PTML-specific survival at 5-, 10-, and 15-years in the training **(A)** and **(B)** validation cohorts.

DCA was performed to compare the clinical utility and benefits of the nomogram with that of the traditional Ann Arbor staging system. As shown in **Figure 6**, the 5-, 10-, and 15-year DCA curves for the nomogram had a greater risk-of-death net benefit in the training and validation cohorts compared with the Ann Arbor Stage model.

## Discussion

BC-PTML is part of a relatively minor group of systemic malignant lymphomas. The development of effective treatment and management strategies has been more challenging for PTML than for primary malignant lymphoma at other sites. Thus, a model to predict CSS in patients with BC-PTML could facilitate the selection of appropriate treatment options and management strategies for these patients. The Ann Arbor staging system helps to predict outcomes for patients with BC-PTML but does not consider some important risk factors such as age and treatment modality. The present study combined multiple risk factors to construct a more comprehensive model for improved prediction of CSS in patients with BC-PTML. Compared with the traditional Ann Arbor staging system, the nomogram diagnostic model with six variables, including radiation, surgery, Ann Arbor Stage, chemotherapy, histological subtype, and age at diagnosis, more accurately assessed and predicted CSS in BC-PTML patients in both a training and a validation cohort.

Considering the influence of the risk factors described above, the conventional Ann Arbor staging system may not be a good predictor of CSS in patients with BC-PTML. Thus, a nomogram was developed to predict BC-PTML patient CSS by combining the above factors. To the best of our knowledge, this is the first comprehensive and detailed nomogram model constructed to predict CSS in a large population of patients with BC-PTML. Prior studies have found that most patients with PTML are women, with a male: female prevalence ratio of 1:3 (3). In the 1,152 BC-PTML patients in the current study, the male: female ratio was 1:2. The prevalence ratio may be related to differences in the level of hormone secretion between the two sexes. However, while the prevalence differed between the sexes, this variable was not an independent prognostic factor for CSS in BC-PTML patients after log-rank and single multivariate Cox analysis. The present study found that age was an independent prognostic factor affecting CSS in patients with BC-PTML and that the prevalence of DLBCL was higher compared with that of MALT, and both these findings are consistent with previous observations (15). Hashimoto’s thyroiditis is thought to be a risk factor for primary thyroid lymphoma, especially the MALT subtype (15, 16), but the SEER database does not indicate whether or not a patient has this disease.

Treatment of primary thyroid lymphoma depends on the pathological subtype and the stage of the disease (17). While prior studies indicated that radiotherapy alone or single chemotherapy should be used to treat thyroid MALT and combination chemotherapy should be used to treat DLBCL, the sample sizes in these studies were small (18–20). The nomogram constructed in the current study found that radiotherapy did not significantly impact CSS in BC-PTML patients and chemotherapy was an independent protective factor, which is consistent with the findings of Vardell et al. (21). Some studies have suggested that chemotherapy combined with radiotherapy is an effective treatment for patients with DLBCL (22). Thus, the efficacy of radiotherapy for patients with BC-PTML requires additional analysis.

There are conflicting ideas about the role of surgical treatment in patients with BC-PTML (23). Among patients with advanced tumors that could not be completely removed, the prognosis was worse for those who received surgery followed by adjuvant chemotherapy compared with those who received a combination of open biopsy and chemotherapy (24). More aggressive guardianship resection is not as effective as modern radiotherapy at promoting complete remission and disease-free survival (25). Mack et al. concluded that surgery could be used to improve survival for patients with MALT at specific stages (26). The current study found that the coefficient of surgical treatment was not statistically significant in the nomogram model. Consequently, further study may be required to determine the effect of surgical treatment on CSS in patients with BC-PTML.

The ability of the newly established nomogram to predict CC in patients with BC-PTML was compared with the conventional Ann Arbor staging system and was found to have improved discrimination and predictive ability. In addition, DCA analysis demonstrated that the model developed in the current study was more accurate compared with the conventional staging approach. Several related studies have used DCA curves to verify the net benefit and clinical utility of a model’s predictive ability. The new nomogram model developed here may help clinicians quantify the risk of CSS for cancer patients and may inform the design of more effective individualized treatment and management strategies.

This study does have some limitations. First, it is a retrospective study that uses data from the SEER database, which may have some built-in biases. However, patients with incomplete information were excluded from the study to help limit bias. Second, many important factors associated with CSS in patients with BC-PTML were not included in the SEER database, such as a history of Hashimoto’s thyroiditis. Finally, the model was validated internally using the leave-out method, and external validation is still required to assess the efficacy of the model.

In summary, a novel nomogram was constructed and validated to predict 5-, 10-, and 15-year CSS in patients with BC-PTML using SEER data. The model included six variables—radiation, primary site of surgery, Ann Arbor Stage, chemotherapy, histological subtype, and age at diagnosis. After validation using multiple methods, the nomogram was compared with the Ann Arbor staging system and found to have good discrimination and a net clinical benefit.

## Data availability statement

The datasets presented in this study can be found in online repositories. The names of the repository/repositories and accession number(s) can be found in the article/**Supplementary Material**.

## Ethics statement

Ethics committee approval was not required for this study because samples were obtained from public databases and private patient information was hidden from the SEER database.

## Author contributions

SJ and XHL were responsible for the conception and design of the study. SJ and LX collected data and did the statistical analysis. CLH and YWY wrote and revised the manuscript, YWY made major contributions to manuscript language editing. All authors have read and approved the final version of the manuscript.

## Conflict of interest

The authors declare that the research was conducted in the absence of any commercial or financial relationships that could be construed as a potential conflict of interest.

## Publisher’s note

All claims expressed in this article are solely those of the authors and do not necessarily represent those of their affiliated organizations, or those of the publisher, the editors and the reviewers. Any product that may be evaluated in this article, or claim that may be made by its manufacturer, is not guaranteed or endorsed by the publisher.
